# Transcriptome Differences in Response Mechanisms to Low-Nitrogen Stress in Two Wheat Varieties

**DOI:** 10.3390/ijms222212278

**Published:** 2021-11-13

**Authors:** Huishu Yan, Huawei Shi, Chengmei Hu, Mingzhao Luo, Chengjie Xu, Shuguang Wang, Ning Li, Wensi Tang, Yongbin Zhou, Chunxiao Wang, Zhaoshi Xu, Jun Chen, Youzhi Ma, Daizhen Sun, Ming Chen

**Affiliations:** 1 Key Laboratory of Sustainable Dryland Agriculture, College of Agriculture, Shanxi Agricultural University, Jinzhong 030801, China; yanhs111@163.com (H.Y.); mrshihuawei@163.com (H.S.); 18392253101@163.com (C.H.); xu1253824860@163.com (C.X.); wsg6162@126.com (S.W.); 13159862006@163.com (N.L.); 2 Institute of Crop Sciences, Chinese Academy of Agricultural Sciences (CAAS), National Key Facility for Crop Gene Resources and Genetic Improvement, Key Laboratory of Biology and Genetic Improvement of Triticeae Crops, Ministry of Agriculture, Beijing 100081, China; caasluomingzhao@163.com (M.L.); tang_wensi@yeah.net (W.T.); zhouyongbin@caas.cn (Y.Z.); wangchunxiao@caas.cn (C.W.); xuzhaoshi@caas.cn (Z.X.); chenjun01@caas.cn (J.C.); mayouzhi@caas.cn (Y.M.)

**Keywords:** wheat, nitrogen stress tolerance, transcriptome, calcium

## Abstract

Nitrogen plays a crucial role in wheat growth and development. Here, we analyzed the tolerance of wheat strains XM26 and LM23 to low-nitrogen stress using a chlorate sensitivity experiment. Subsequently, we performed transcriptome analyses of both varieties exposed to low-nitrogen (LN) and normal (CK) treatments. Compared with those under CK treatment, 3534 differentially expressed genes (DEGs) were detected in XM26 in roots and shoots under LN treatment (*p* < 0.05, and |log2FC| > 1). A total of 3584 DEGs were detected in LM23. A total of 3306 DEGs, including 863 DEGs in roots and 2443 DEGs in shoots, were specifically expressed in XM26 or showed huge differences between XM26 and LM23 (log2FC ratio > 3). These were selected for gene ontology and Kyoto Encyclopedia of Genes and Genomes enrichment analyses. The calcium-mediated plant–pathogen interaction, MAPK signaling, and phosphatidylinositol signaling pathways were enriched in XM26 but not in LM23. We also verified the expression of important genes involved in these pathways in the two varieties using qRT-PCR. A total of 156 transcription factors were identified among the DEGs, and their expression patterns were different between the two varieties. Our findings suggest that calcium-related pathways play different roles in the two varieties, eliciting different tolerances to low-nitrogen stress.

## 1. Introduction

Nitrogen (N) and nitrogen fertilizers play key roles in the growth and development of crops and crop yield, respectively [1]. However, excessive application of nitrogen fertilizers causes serious environmental pollution [2]. Therefore, high nitrogen use efficiency (NUE) is critical for improving crop yield and reducing environmental pollution [3]. In plants, NUE is a complex trait associated with nitrogen accumulation and metabolism, including absorption, transportation, assimilation, and re-mobilization or re-utilization [4,5]. Wheat is one of the most widely planted cereal crops in the world and is also the main source of protein in human food [6]. However, most of the studies on NUE focus on model plants such as *Arabidopsis* or rice. Owing to the complexity of the wheat genome, research on wheat NUE-related regulatory networks remains limited.

Plants usually absorb nitrogen either in inorganic (nitrate and ammonium) or organic (amino acids and urea) form [7]. Rice is a typical ammonium-loving crop [8], whereas wheat absorbs nitrogen mainly in the form of nitrate [6,9]. Enzymes involved in nitrogen assimilation include nitrate reductase (NR), nitrite reductase (NiR), glutamine synthetase (GS), glutamate synthetase (GOGAT), glutamate dehydrogenase (GDH), glutamate decarboxylase (GAD), and asparagine synthetase (ASNS) [10]. The knockout of *TabZIP60* enhances the activity of NADH-dependent GOGAT, which in turn enhances the absorption of nitrogen in wheat [1]. *ARE1* is a crucial negative regulator of the nitrogen-assimilation pathway, and a functional deletion mutant of *TaARE1* has been found to increase yield under nitrogen-limited conditions [11].

Many genes involved in the tolerance of plants to low nitrogen have been identified. There are 53 *nitrate transporter* *1 (NRT1)*/*peptide transporter family* (*NPF*)-like genes and 7 *NRT2*-like genes in *the* *Arabidopsis* genome [12]. Among them, four *NRT2*-like transporters including *NRT2.1*, *NRT2.2*, *NRT2.4*, and *NRT2.5*, and two *NRT1*/*NPF-like* transporters, including *NRT1.1* and *NRT1.2*, are of greater importance for root nitrate uptake than the other members [13,14]. Both *NRT2*-like genes *TaNRT2.1* and *TaNRT2.2* are regulated by the transcription factor *TaWRKY20* in wheat [15]. Nitrate treatment triggers a specific calcium ion (Ca^2+^)–calcium-dependent kinase (CPK) signaling pathway [16]. For example, the *AtCPK10*/*AtCPK30*/*AtCPK32* phosphorylates NIN-LIKE PROTEIN (NLP)-like transcription factor, which causes nuclear localization of NLP and induces the expression of nitrate-responsive genes within minutes following nitrate treatment [16]. Calcium-related proteins including *CBL1*, *CBL9,* and *CIPK23* participate in the regulation of *NRT1.1* functions [12]. For hormone-related pathways, many hormones such as CKs [12], GA [17], auxin [18], and ABA [19] respond to nitrate treatment. Ethylene also regulates the expression of nitrate transporter genes *NRT1.1* and *NRT2.1*, whereas nitrate deficiency induces the expression of ethylene signaling components, including *EIN3* and *EIL1*, and triggers ethylene biosynthesis and the ethylene signaling pathway [20]. More than 40 transcription factors are involved in the transcriptional regulation of nitrate transport, reduction, and assimilation in plants [12]. In rice, a huge regulatory network for nitrogen response, including 1660 interactions between 431 genes, 345 transcription factors, and 98 promoters was completed using a yeast one-hybrid analysis [21].

Transcriptome analysis is a rapid and effective method for studying regulatory networks in crops [22,23]. A transcriptome analysis of two rice varieties with different genotypes under different nitrogen conditions showed that most differentially expressed genes (DEGs) are associated with starch and chloroplast metabolism and signal transduction [24]. The re-application of nitrate to nitrogen-deprived *Arabidopsis* significantly changes the expression of pathway genes in primary and secondary metabolism, cell growth, hormone response, protein synthesis, signal transduction, and transcriptional regulation [25]. A transcriptome analysis of low-nitrogen-treated wheat seedlings revealed that carbon and nitrogen metabolism, antioxidant processes, and environmental adaptation-related pathways are involved in the low-nitrogen stress response [26].

Here, to understand the molecular mechanism of low-nitrogen stress response using low-nitrogen-tolerant wheat varieties, to screen more nitrogen-related genes, and to decipher the basis for nitrogen-efficient breeding, we screened two wheat varieties resistant or sensitive to low nitrogen through chlorate sensitivity experiments. The DEGs of the two wheat varieties in response to low-nitrogen stress were identified. We also identified three pathways that may be important in response to low-nitrogen stress in wheat, namely the calcium-mediated plant–pathogen interaction, MAPK signaling, and phosphatidylinositol signaling pathways.

## 2. Results

### 2.1. Screening of Wheat Varieties Based on Chlorate and Low-Nitrogen Stress-Tolerance Analyses

**Figure 1:**
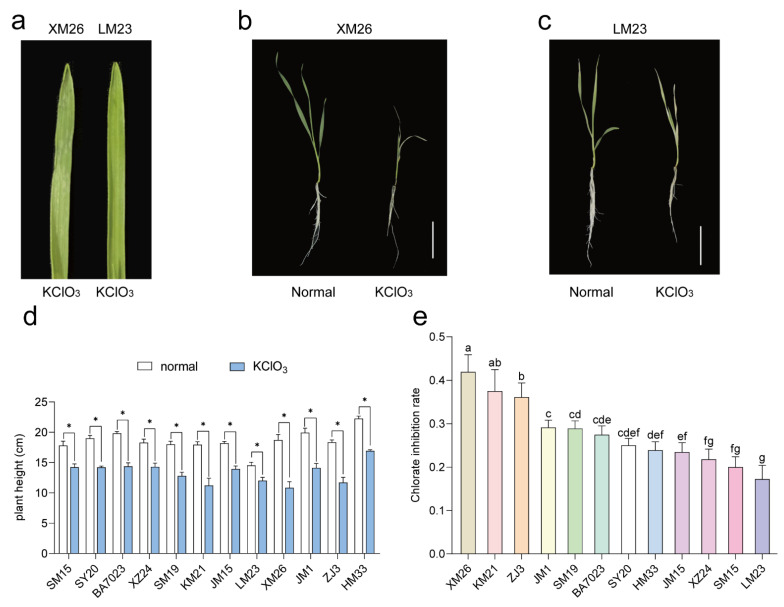
Chlorate tolerance analysis of 12 wheat varieties during the seedling stage. (**a**) Shapes of the shoots of XM26 and LM23 after KClO_3_ treatment for 20 h. (**b**,**c**) Phenotype of whole XM26 and LM23 plants under normal conditions (**left**) and KClO_3_ treatment (**right**) for 4 d. (**d**,**e**) Plant heights and chlorate inhibition rates in different wheat varieties treated with 0 (normal) and 4 mmol/L KClO_3_ for 4 d. * indicates a significant difference (*p* < 0.05) between different treatments. Lowercase letters indicate significant differences (*p* < 0.05) between different wheat varieties.

Chlorate (ClO_3_^−^), a nitrate analog, shares the same absorption and assimilation pathways as nitrate, whereas chlorate becomes toxic when it is reduced to chlorite. Therefore, chlorate sensitivity can be used for phenotypic identification to mimic plant uptake or to assimilative activity for nitrate, providing rapid identification of differential phenotypes [27,28]. To screen the wheat varieties resistant to low-nitrogen stress, we conducted a chlorate-tolerance analysis of 12 wheat varieties and found that the plant height in all wheat varieties was reduced after treatment with potassium chlorate (KClO_3_). The growth inhibition rate indicated that the wheat variety with the highest inhibition rate of chlorate was XM26 and that the wheat variety with the lowest inhibition rate of chlorate was LM23 (Figure 1).

**Figure 2:**
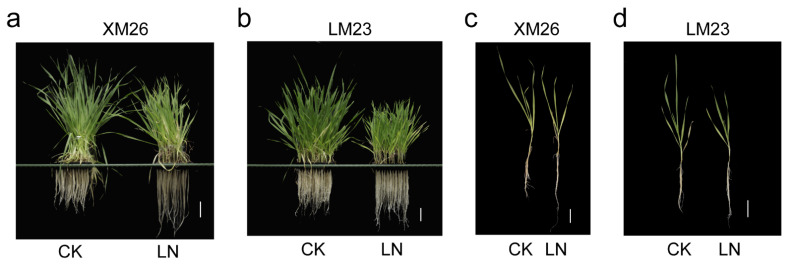
Growth phenotypes of XM26 and LM23 under normal (CK) and low-nitrogen (LN) conditions. (**a**,**c**) The phenotype of XM26 after CK and LN treatments for 23 d. Bar = 5 cm. (**b**,**d**) Phenotype of LM23 following CK and LN treatments for 23 d. Bar = 5 cm.

Simultaneously, we performed a low-nitrogen stress-resistance analysis of the two abovementioned wheat varieties. Compared with the normal (CK) condition, the shoot height, leaf length, dry weight, N content, and N accumulation in both wheat varieties decreased; however, root elongation increased under low N treatment (LN; Figure 2; Table S1). The analysis showed that the values of plant height, leaf length, shoot N accumulation, root N content, and root N accumulation were significantly different between XM26 and LM23 under CK conditions. Following LN treatment, the values of root length, plant height, leaf length, shoot dry weight, and shoot N content were significantly different between XM26 and LM23. We also compared each value for wheat varieties under LN and CK conditions and found that all ratios of XM26 were higher than those for LM23, suggesting that XM26 is more resistant to low-nitrogen stress than LM23 (Table S1).

### 2.2. Transcriptome Analysis of the Two Wheat Varieties under Normal and Low-Nitrogen Conditions

The roots and shoots of both wheat varieties were harvested at 12 h after CK and LN treatments. A transcriptome sequence was subsequently completed using the Illumina HiSeq platform. The number of clean reads for each sample varied from 58.35 million to 78.05 million, with a mean of 65.20 million. The content of Q20 for each sample varied from 97.92% to 98.56%, with a mean of 98.35%. The content of Q30 for each sample varied from 93.84% to 95.42%, with a mean of 94.90%. The GC content (Base G + Base C) for each sample varied from 49.68% to 54.19%, with a mean of 52.06%. The 88.61% and 70.71% reads from the shoot and root samples were uniquely mapped. Based on these quality control data, the transcriptome data were deemed suitable for subsequent analyses.

**Figure 3:**
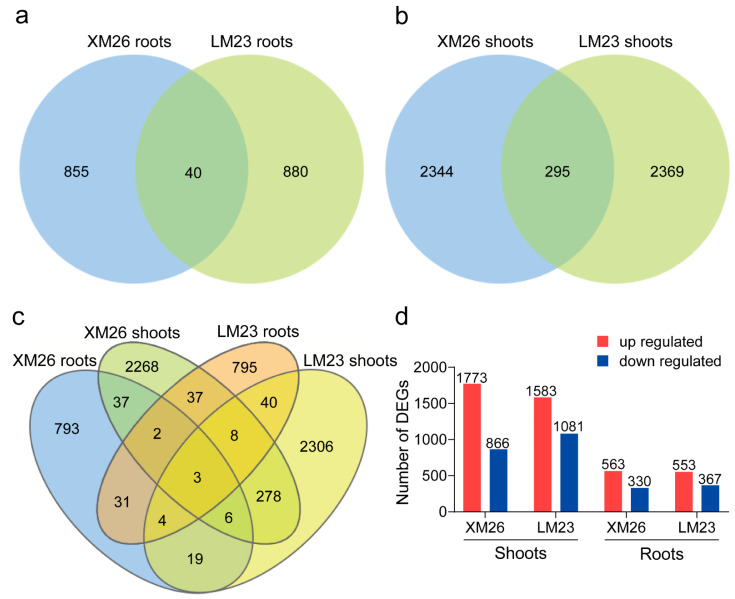
Differentially expressed genes (DEGs) between the two wheat varieties at the seedling stage under low nitrogen (LN) relative to control (CK) conditions. (**a**–**c**) Venn diagrams of DEGs in roots and shoots of XM26 and XM23. (**d**) Histogram showing the number of DEGs in roots and shoots of XM26 and LM23. Red and blue represent upregulated and downregulated genes, respectively.

The transcriptional levels were normalized using the FPKM method. Genes with *p* < 0.05, and |log2(fold change)| > 1 were defined as DEGs. Consequently, 2639 DEGs were identified in the shoots of XM26 under LN conditions compared with the CK and included 1773 and 866 upregulated and downregulated genes, respectively. Among the DEGs of XM26, there were 895 DEGs in the roots, including 565 and 330 upregulated and downregulated genes, respectively. We identified 2664 DEGs in LM23 shoots following LN treatment compared with the genes in CK, including 1583 and 1081 upregulated and downregulated genes, respectively. A total of 920 DEGs were identified in LM23 roots, including 553 upregulated and 367 downregulated genes (Figure 3d; Table S2). A Venn diagram analysis showed that 295 DEGs were commonly identified in shoots between XM26 and LM23, and 40 DEGs were commonly identified in roots (Figure 3a–c). In addition, 855 DEGS were specific to XM26 and 880 were specific to LM23 in the roots. In shoots, 2344 DEGs were specific to XM26 and 2369 were specific to LM23 (Figure 3a–c). Among all DEGs, three were significantly expressed in the roots and shoots of the two wheat varieties simultaneously.

To verify the accuracy of RNA-Seq data, we randomly selected eight DEGs for further qRT-PCR, including ethylene-responsive transcription factor *ERF053*, chitin-inducible gibberellin-responsive protein 1 (*CIGR 1*), calmodulin-binding protein 60 B-like (*CBP60*), CDP-diacylglycerol-serine O-phosphatidyltransferase 2 isoform X2 (*PSS2*), group II HKT transporter (*HKT9*), serine/threonine-protein kinase *SAPK3*, DIBOA-glucoside dioxygenase BX6-like (*BX6*), and transcription factor MYB108-like *(MYB108*). The qRT-PCR and RNA-Seq findings were highly consistent, indicating the reliability of the RNA-Seq data (Figure S1).

### 2.3. GO and KEGG Enrichment Analyses of DEGs

**Figure 4:**
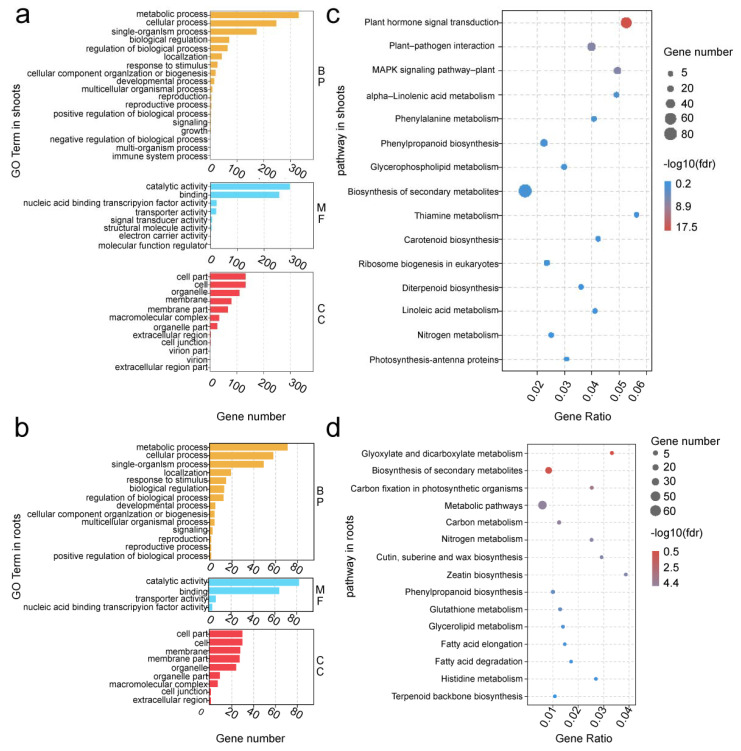
Gene ontology (GO) and Kyoto Encyclopedia of Genes and Genomes (KEGG) enrichment analysis of differentially expressed genes (DEGs). GO enrichment analysis of DEGs in shoots (**a**) and roots (**b**). BP represents biological processes. MF represents molecular functions. CC represents cellular components. KEGG enrichment analysis of DEGs in shoots (**c**) and roots (**d**).

From 5008 DEGs in shoots and 1775 DEGs in roots, we selected DEGs specifically expressed in XM26, DEGs commonly expressed, and the ratio of log2 (fold change) value > 3 between the two wheat varieties for further analysis. We performed a gene ontology (GO) enrichment analysis of a total of 2443 DEGs in shoots and 863 DEGs in roots. We classified DEGs into three categories: biological processes (BP), molecular functions (MF), and cellular components (CC). In shoots, the GO terms of the metallic, cellular, and single-organic processes were significantly enriched for BP. The significantly enriched GO terms in MF were catalytic activity, binding, and nucleic acid binding transcription factor activity. The significant enrichment terms in CC were cell, cell part, and organelle. In roots, the significant enrichment terms in BP were metabolic, cellular, and single-organism processes. The significant enrichment terms in MF were catalytic activity, binding, and transporter activity. The GO terms, including the cell, cell part, and membrane, were significantly enriched in CC (Figure 4a,b). DEGs were mapped using different KEGG pathways. The results showed that pathways such as plant hormone signal transduction, plant–pathogen interaction, MAPK signaling pathway–plant, alpha-Linolenic acid metabolism, and phenyl orchid metabolism were significantly enriched in shoots. Pathways including glyoxylate and dicarboxylate metabolism, biosynthesis of secondary metabolites, carbon fixation in photosynthetic organizations, metabolic pathways, carbon metabolism, and others were also significantly enriched (Figure 4c,d).

### 2.4. Validation of Some Key Genes Involved in Important Pathways

**Figure 5:**
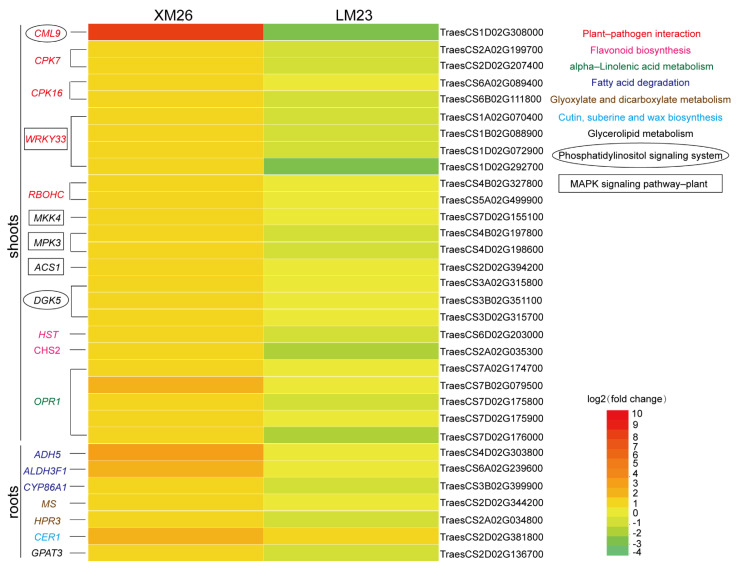
Heatmap of the expression of differentially expressed genes (DEGs) involved in important pathways. The color scale indicates gene expression levels, and the number represents log2 (fold change) values. Red represents high fold change; green indicates low fold change. The left y-axis indicates gene name, and the right y-axis indicates gene ID. Different colors and shapes of gene name indicate different pathways labeled in the top right corner.

**Figure 6:**
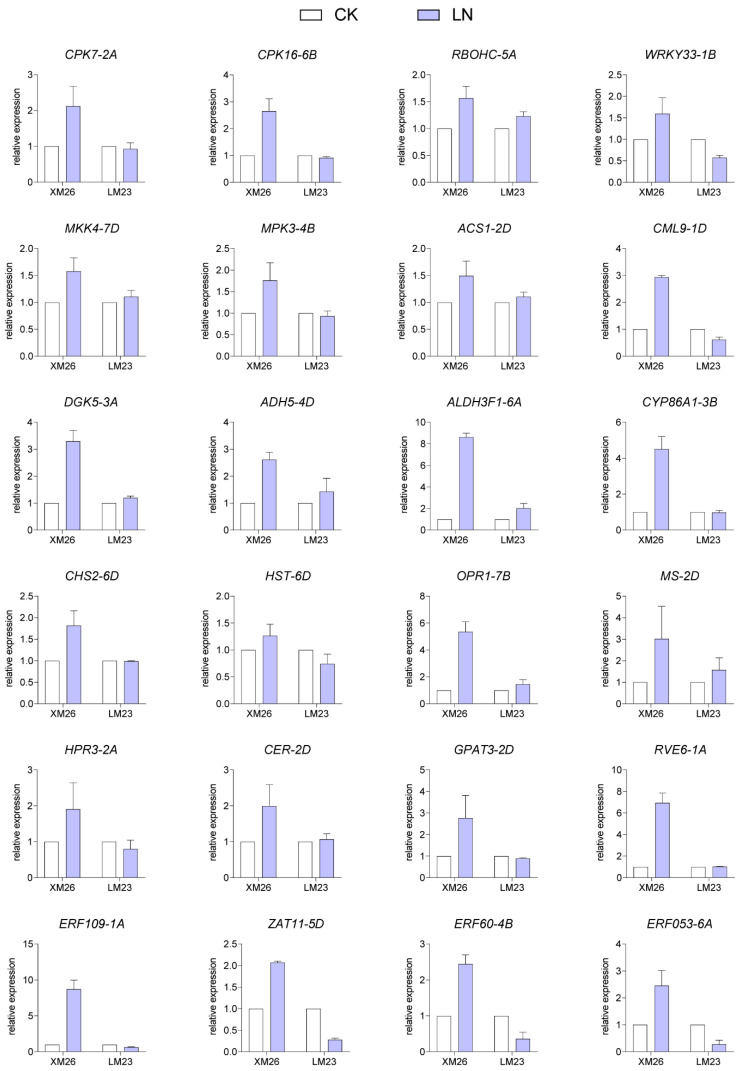
Some important differentially expressed genes verified using qRT-PCR. Seedlings (7 d old) of wheat were grown hydroponically in a nutrient solution under normal (CK, 2 mM N) or low-nitrogen (LN, 0.2 mM N) treatments for 12 h. White represents CK treatment, and blue represents LN treatment. Error bars indicate ± SD (*n* = 3).

We found that the expression profiles of many genes belonging to pathways including the plant–pathogen interaction pathway; mitogen-activated protein kinases (MAPK) signaling pathway; phosphatidylinositol signaling pathway; fatty acid degradation pathway; flavonoid biosynthesis; alpha-linolenic acid metabolism; glyoxylate and dicarboxylate metabolism; glycerolipid metabolism; and cutin, suberine, and wax biosynthesis were significantly different between XM26 and LM23, suggesting that these genes may be related to different tolerances to low-nitrogen stress for the two wheat varieties (Figure 5). Genes such as *CALM* (*CLM9)*, *CDPK* (*CPK7*, *CPK16*), *RbOH* (*RBOHC*), and *WRKY33* belonging to the plant–pathogen interaction pathway were upregulated in XM26 shoots; however, there was no significant difference in LM23 (Figure 5). Interestingly, these genes also belong to the calmodulin-related genes. These genes were verified by qRT-PCR, and the results were consistent with those of RNA-Seq analysis (Figure 6). Transcriptome data showed that the MAPK pathway-related genes *MKK4*, *MPK5*, and 1-aminocyclopropane-1-carboxylate synthase 1/2/6 *ACS1* were upregulated in XM26 in shoots (Figure 5). In LM23, only *MKK4* was upregulated, which was verified by qRT-PCR (Figure 6). In the phosphatidylinositol signaling pathway, when the cells were stimulated to produce IP_3_ and the concentration of Ca^2+^ was increased [29], the calcium-related protein *CML9* was significantly upregulated in XM26 in shoots; however, there was no significant difference in LM23 (Figure 6). Similarly, diacylglycerol kinase *DGK5*, produced by diacylglycerol (DG), was also upregulated in XM26 but its level unchanged in LM23. Therefore, we speculate that the Ca^2+^-mediated phosphatidylinositol signaling pathway responds to LN-stress, and different expressions of *DGK5* and *CML9* might have different tolerances between XM26 and LM23 (Figure 6). Transcriptome analysis showed that fatty acid degradation pathway-related genes, including *ADH5*, *ALDH3F1*, and *CYP86A1*, were significantly upregulated in XM26; however, there was no significant difference in LM23 roots (Figure 5 and Figure 6), suggesting that the fatty acid degradation process might be involved in the different responses to low-nitrogen stress between the two wheat varieties.

The flavonoid biosynthesis-related gene *HST* was significantly upregulated in the shoots of XM26 but showed no significant difference in LM23. Another flavonoid biosynthesis-related gene *CHS2* was significantly upregulated in XM26 but was downregulated in LM23 (Figure 5 and Figure 6). The alpha-linolenic acid metabolism gene *OPR1* was significantly upregulated in XM26, but its expression was not significantly different in LM23 (Figure 5 and Figure 6). In the roots, glyoxylate and dicarboxylate metabolism-related genes *MS* and *HPR3* were significantly upregulated in XM26 but showed no significant difference in LM23 (Figure 5 and Figure 6). Cutin, suberine, and wax biosynthesis-related gene *CER1* was significantly upregulated in XM26 but showed no significant difference in LM23 (Figure 5 and Figure 6). The glycerolipid metabolism-related gene *GPAT3* was significantly upregulated in XM26 but showed no significant difference in LM23 (Figure 5 and Figure 6).

### 2.5. Transcription Factors among DEGs

Among the DEGs, there were 132 transcription factor genes in shoots (14 DEGs were co-expressed in XM26 and LM23, and the other 118 DEGs were specifically expressed in XM26 or LM23), including 35 WRKY, 29 ERF, 26 BHLH, 18 zinc, and 24 transcription factors belonging to other families. A total of 24 transcription factors were found among root DEGs (four DEGs were co-expressed in XM26 and LM23, and the other 20 DEGs were specifically expressed in XM26 or LM23). These DEGs included eight zinc, five ERF, four WRKY, and seven other family transcription factors (Figure S2). The expression patterns of the above 156 transcription factor genes were significantly different between the two wheat varieties. Among the 156 transcription factor genes, we verified the differences in the expression of five in the two wheat varieties using qRT-PCR (Figure 6).

## 3. Discussion

### 3.1. Calcium-Mediated Pathways Help Regulate Low-Nitrogen Stress Response between XM26 and LM23

We found that MAPK pathway-related proteins such as MPK3 and ACS1 were significantly upregulated in XM26, whereas the expression of these genes was not different in LM23, suggesting that the MAPK pathway was enhanced in resistant wheat varieties under low-nitrogen stress (Figure 5 and Figure 6). MAPK-related pathways are reported to be involved in plant responses to low-nitrogen stress [30]. The wheat MAPK gene *TaMPK14* plays an important role in regulating low-nitrogen stress tolerance in plants, and *TaMPK14* overexpression in tobacco showed higher NUE [31]. *AtMPK3* is significantly induced by low-temperature stress in *Arabidopsis* [32]. Therefore, we believe that the MAPK signaling pathway is involved in the low-nitrogen tolerance of different wheat varieties. In our study, the calcium pathway-related genes *CML9* and *DGK5* were significantly upregulated in XM26 and were unchanged in LM23 (Figure 5 and Figure 6). Calcium-dependent kinases (CPKs), downstream of calcium signaling, act as a link between NRT1.1 and NLP-TFs [33]. The transport activity of NRT1.1 induces calcium waves through the action of phospholipase C and inositol triphosphate (IP_3_) [33]. The phosphatidylinositol signal system produces two second messengers, IP_3_ and diacylglycerol (DG), which convert extracellular signals into intracellular signals [29]. *AtCML9* in the phosphatidylinositol signaling system is involved in salt-stress tolerance through its effect on the ABA-mediated pathway [34]. *DGK5* promotes responses to cold in Arabidopsis [35]. Therefore, we suggest that the Ca^2+^ pathway-related CML9 and DGK5 may be involved in the regulation of response to low-nitrogen stress in different wheat varieties.

**Figure 7:**
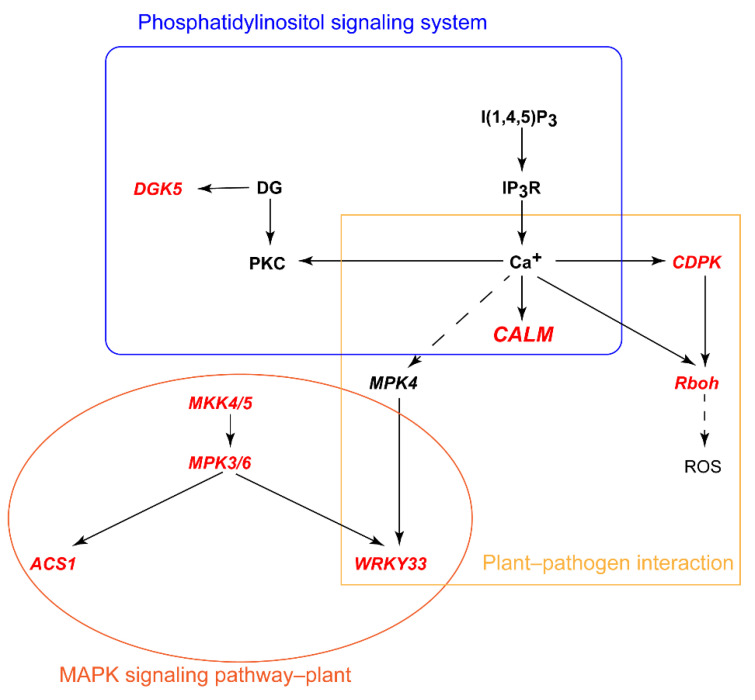
Expression patterns of differentially expressed genes (DEGs) in multiple calcium ion-mediated pathways. Red indicates DEGs with a significant difference in XM26. Different shapes represent different pathways.

We found other Ca^2+^-related genes, such as *CPK7*, *CPK16*, *CML9*, *RBOHC*, and *WRKY33*, in the plant–pathogen interaction pathway (Figure 5 and Figure 6). *AtWRKY33* overexpression in plants has been shown to be associated with a better NUE [36]. Many genes involved in plant–pathogen interaction pathways are also involved in abiotic stress responses. NO_3_^−^ can trigger specific Ca^2+^–CPK signals, and AtCPK10/AtCPK30/AtCPK32 phosphorylates the NLP transcription factor, leading to its nuclear localization, and induces nitrate-responsive gene expression within minutes following nitrate treatment [16]. Calcium-related proteins (*CBL1*, *CBL9*, and *CIPK23*) participate in the regulation of NRT1.1 function [12]. In *Arabidopsis*, nitrate triggers a unique Ca^2+^-CDPK signal through six CPKac (CPK7ac, CPK8ac, CPK10ac, CPK13ac, CPK30ac, and CPK32ac) [37]. Calcium is an important secondary messenger in nitrate signaling [16]. We found that Ca^2+^ participates in the plant–pathogen interaction pathway and phosphatidylinositol signaling system. Interestingly, MPK3 was previously characterized as being upstream of the transcription factor WRKY33 [36]. Thus, we suggest that calcium-mediated pathways play important roles in regulating the low-nitrogen stress response in different wheat varieties (Figure 7).

### 3.2. Screening Low-Nitrogen-Tolerance Wheat Varieties Using the Chlorate Tolerance Analysis

Chlorate treatment can significantly reduce the plant height in rice. Following a chlorate sensitivity test on 134 rice varieties, indica rice was found to be phenotypically distinct from japonica rice owing to its significantly higher chlorate sensitivity [38]. Similar results were observed in our study, wherein the plant height was significantly lower after 4 mmol/L KClO_3_ treatment than under normal treatment (Figure 1). The low-nitrogen-tolerant variety XM26 and the low-nitrogen-intolerant variety LM23 were screened out. In this study, the plant height, leaf length, dry weight, fresh weight, total nitrogen content, and nitrogen accumulation were reduced to different degrees following 23 d of low-nitrogen treatment. The root length also exhibited different degrees of elongation, consistent with previous studies (Figure 2). The results also showed that XM26 is more tolerant to low nitrogen than LM23, further supporting the reliability of the chlorate-tolerance-screened varieties (Table S1). In conclusion, chlorate was used to screen wheat varieties with low-nitrogen tolerance. The mechanism underlying the low-nitrogen tolerance of wheat varieties with low-nitrogen tolerance under LN stress was clarified by transcriptome sequencing. Some of the genes involved in resistance to LN were screened out, which provides useful information for future research on the molecular breeding of high-nitrogen-efficiency wheat.

## 4. Materials and Methods

### 4.1. Plant Materials and Growth Conditions

The wheat materials used in this experiment were obtained from the Chinese Academy of Agricultural Sciences. Wheat seeds were sterilized with 2% H_2_O_2_ for 30 min and germinated in distilled water at room temperature [39]. Three d later, they were transplanted into black 96-well plastic boxes (126 mm × 85 mm × 110 mm) and placed in light incubators. The light/dark conditions were 14/10 h, and the corresponding temperatures were 22/18 °C [5]. At the one-leaf one-heart period (7 d old), they were cultured with an improved Hoagland nutrient solution and subjected to CK or LN treatments [40]. The pH of the solution was adjusted to 6.0, using NaOH or HCl, where N was provided by Ca(NO_3_)_2_, and 1 mM Ca(NO_3_)_2_ was used in the CK treatment. An amount of 0.1 mM Ca(NO_3_)_2_ was used in the LN treatment, and CaCl_2_·2H_2_O was used to supplement Ca and to maintain its concentration at 2.5 mM. After 30 d, the phenotype and nitrogen content were determined.

### 4.2. Chlorate Sensitivity Experiment

The toxicity experiment was conducted using 4 mM KClO_3_ instead of Ca(NO_3_)_2_, and the chlorate toxicity experiment was conducted in a one-leaf one-heart period. After 4 d, the seedling height was measured and the chlorate inhibition rate was calculated according to the following formula: chlorate inhibition rate on plant growth = (control treatment height − chlorate treatment height)/control treatment height × 100% [38].

### 4.3. Transcriptome Analysis under Normal and Low-Nitrogen Conditions

For RNA-Seq samples, XM26 and LM23 seeds were grown under the above conditions and samples were collected after treatment with LN (0.2 mM) and CK (2 mM) for 12 h, respectively. In a preliminary experiment, we found that approximately 12 h after chlorate treatment, the phenotype of XM26 began to change (the leaf tip began to wilt), and there was a significant difference between the two varieties at 20 h (Figure 1a). Therefore, we surmise that a considerable number of genes responding to nitrogen were activated at 12 h. The samples were immediately frozen in liquid nitrogen and stored at −80 °C. All samples (a total of 24 samples from 2 genotypes (XM26 resistant to low nitrogen and LM23 not resistant to low nitrogen) × 2 treatments (LN and CK) × 2 tissues (roots and shoots) × 3 biological repetitions) were prepared for further RNA-Seq analysis.

RNA isolation, cDNA library construction, and Illumina sequencing were performed with the assistance of Genedenovo Biotechnology (Guangzhou, China). Clean reads were obtained by removing adapter-containing, N-containing greater than 10%, all A-base, and low-quality reads from the raw data. The clean reads were mapped to a cDNA database from the wheat variety Chinese Spring (IWGSC_RefSeq_v1.1). The expression level of each gene was measured using the number of fragments per kilobase per million reads (FPKM). Differences in the RNA transcript levels were analyzed using DESeq2 and edgeR. *p* < 0.05, and |log2 (fold change)| > 1 were set as the thresholds for identifying DEGs [41]. GO enrichment analysis of differential genes was performed based on the GO database (http://www.geneontology.org/, accessed on 9 November 2021), and pathway enrichment analysis was performed based on the KEGG database (https://www.kegg.jp/, accessed on 9 November 2021) [42,43]. All raw reading sequences were uploaded in NCBI’s sequence read archive (SRA) under the accession number PRJNA773211.

### 4.4. RNA Extraction and Quantitative Reverse-Transcription PCR

Total RNA was extracted using the KKFast Plant RNApure Kit (ZP405K-2, Zoman Biotech, Beijing, China), and RNA was reverse transcribed to cDNA (AT311-03, Transgen Biotech, Beijing, China) using the TransScript One-Step gDNA Removal and cDNA Synthesis SuperMix kit. The quantitative reverse transcription polymerase chain reaction (qRT-PCR) was performed using TaqPro Universal SYBR qPCR Mastermix (Q712-02, Vazyme, China). The qRT-PCR conditions were 30 s at 95 °C, followed by 40 cycles of 95 °C for 10 s and 58 °C for 30 s. Relative gene expression was calculated using the 2^−ΔΔT^ method. The experiments were performed in triplicate. The primers used in this study are listed in Table S1.

### 4.5. Statistical Analysis

The seedling height, root length, leaf length, dry weight, N content, and N accumulation were measured. Each dry weight value is the total dry weight of the four seedlings. The nitrogen content was determined using the Kjeldahl method [44]. Following 4 d of chlorate treatment, the seedling height was measured. Morphological data were analyzed using IBM SPSS 23 (Microsoft, Redmond, WA, USA) and displayed in GraphPad Prism 8.0 (GraphPad Software, La Jolla, CA, USA). Significant differences were analyzed using one-way ANOVA in SPSS (www.spss.com, accessed on 9 November 2021). Significance was set at *p* < 0.05.

## 5. Conclusions

In this study, two wheat varieties with different low-nitrogen tolerances were screened through a chlorate sensitivity test, and DEGs were identified in these varieties in response to low-nitrogen stress. We speculate that three pathways, namely the calcium-mediated plant–pathogen interaction pathway, the MAPK signaling pathway, and the phosphatidylinositol signal pathway, might be important in the response of wheat to low-nitrogen stress. These results provide useful information for future research on molecular breeding of high-nitrogen-efficiency wheat.

## Data Availability

The data that support the findings of this study are available from the corresponding author upon reasonable request.

## Supplementary Materials

The following are available online at https://www.mdpi.com/article/10.3390/ijms222212278/s1, Figure S1: RNA-Seq data accuracy verification, Figure S2: Heatmap expression profile of DEGs involved in transcription factors, Table S1: Growth performances of two wheat varieties XM26 and LM23 at 23 d after low N Treatment, Table S2: The primers used for qRT-PCR analysis.
