# Supplementary material for: Transcriptome Differences in Response Mechanisms to Low-Nitrogen Stress in Two Wheat Varieties
Source: Int J Mol Sci. 2021 Nov 13;22(22):12278. doi: 10.3390/ijms222212278 (PMC8622133; doi:10.3390/ijms222212278)
Supplement: Supplementary file 1 [file ijms-22-12278-s001.zip › ijms-1448531-supplementary.pdf]

International Journal of Molecular Sciences

Table S1. Growth performances of two wheat varieties XM26 and LM23 at 23 d after low N Treatment.

Trait	XM26			LM23		
	CK	LN	Relative	CK	LN	Relative
Root length (mm plant^{-1})	154.83	211.83*	1.37	164.42	187.83	1.14
Plant height (mm)	410.58*	312.00*	0.76	302.83	218.97	0.72
Leaf length (mm plant^{-1})	289.25*	235.08*	0.81	218.33	159.75	0.73
Root dry weigh (mg plant^{-1})	112.80	111.20	0.99	128.73	107.40	0.83
Shoot dry weigh (mg plant^{-1})	589.37	385.93*	0.65	511.27	305.87	0.60
Shoot N content (%)	3.62	1.83*	0.51	3.15	1.51	0.48
Shoot N accumulation (mg plant^{-1}DW)	11.37*	5.52	0.49	9.98	4.67	0.47
Root N content (%)	1.83*	1.62	0.88	2.13	1.59	0.75
Root N accumulation (mg plant^{-1}DW)	5.43*	3.81	0.70	6.55	3.90	0.60

CK: Normal condition N level (2 mM N); LN: Low N level (0.2 mM N); Relative: LN/CK. For each line, * indicated a significant difference between the different genotypes of the same treatment (P, 0.05).

Table S2. The primers used for qRT-PCR analysis.

Gene ID	Primer sequences (forward)	Primer sequences (reverse)
ERF053-6B	GTGGAAGGACTAGACACGCA	CCCATCTTTGTCCCAGCCTA
CIGR1-2B	CCGCTGAGCTCGTATGTGAA	CTGAGCAACTGTCAGTGCCA
CBP60-3A	GAACTGTACCGGAAGCACTAC	CATTATCGCGCGCAGTATTTC
PSS2-1B	TGTAGCCATATGCATAGTGGAG	TCAGAGATTCTTTCTGCGGAAT
HKT9-7B	TGCCAATTTTTCTCAGTTCTGCA	AGCGAGGACTATAGTTAGGGT
SAPK3-1A	TGCTCTGAGTTGGTGCTTCT	TGATCTCCCTCTGCACATTCT
BX6-2B	GAGAGTTCATCGCAACGTTTAA	CACGGTTCTGACACTTTACTTG
MYB108-3D	CAGAAGCACGCCAAGCAG	CTGCCGACGGTCATGACAA
CPK7-2A	AATCTGGGTGAGACAGTTAAGG	GGCCAAGTTTATTCAGTCCAAG
CPK16-6B	CACGGGCTTAAAGGGATCCA	CCTCGCCGATTCTCACAGAG
RBOHC-5A	CTACTGCACTAGTGTCTACGAG	GTCTTTCTCGAGAAATCTTGCG
WRKY33-1B	CTCTCCCAACCCAAGTCTCAAT	TGAACGTGAACCCTGCGTG
MKK4-7D	AGAAGCAGTGCCATCATATGAT	CCTCTATCCAACCAGCATATGT
MPK3-4B	CCAACCAAGAACTCTCAGAAGA	GAAGTCGCAGATCTTGAGGTC
ACS1-2D	GAAACCGGAGAGCGCAGAT	GATCAGGTCCAGCGAGAGTT
CML0-1D	GTGCCATGTTTCTTGATTTCCT	TATACATATTGCAGCCAGAGCA
DGK5-3A	CAGGTAGCAAGCTCAAATGGA	CCTTTCCACGTTGCTGTAGAT
ADH5-4D	GCAATTCGGAGGCGTGAAG	CGGCAGCAATGAAACCCAAT
ALDH3F1-6A	GTGTAGTCAATCATGCTCGATG	TGGTTCAGGCATTTTTCACAAG
CYP86A1-3B	GAGGTTTCTGTTCCCTAGCTTC	GATGGTCTCCGTCATGAACTG
CHS2-6D	GGCAACATGTCAAGCGCAT	GCAAACTTGGGTGTAGCAGC
HST-6D	ATGGACCTCAAGCGCCTTATC	ACTTGAAGTGGGTCACCTG
OPR1-7B	GTCTCCACCAACGATTTTCAAC	CGTCTAAAGTCATCGACGATCA
MS-2D	CAAGCTCTGACTGGGAATCTAG	AGCAACACAGCCAATATAGGTA
HPR3-2A	TCTGTCCTCCAAAGCAGCAG	GTCGTGTTCTCCATCGCTCC
CER-2D	GGGTCAGAGTGCTTAGTCTAGG	GGAACCCGCAATTTAAGCATGT
GPAT3-2D	CCGACGCCATGGACGAAAA	GACGGTGATCGGGTCTAGGA
RVE6-1A	CTCAGTTCATCACACTTCACTG	GCCATCGTCTTCTTCTCTCTAT
ERF109-1A	CATCATCGCTTGAAACGACAAA	GTTCCTACTCCTGCTGATTGAG
ZAT11-5D	TCCATTGTTCCTCCTACCGA	AAACAATCGGCTCCTCCTCC
ERF060-4B	GTTCCTACTCCTGCTGATTGAG	CTGTCCACCATGTGATACATCG
ERF053-6A	AGCCTCTCTCGTTCGCCA	CGCCACATCCATTCCAACAA

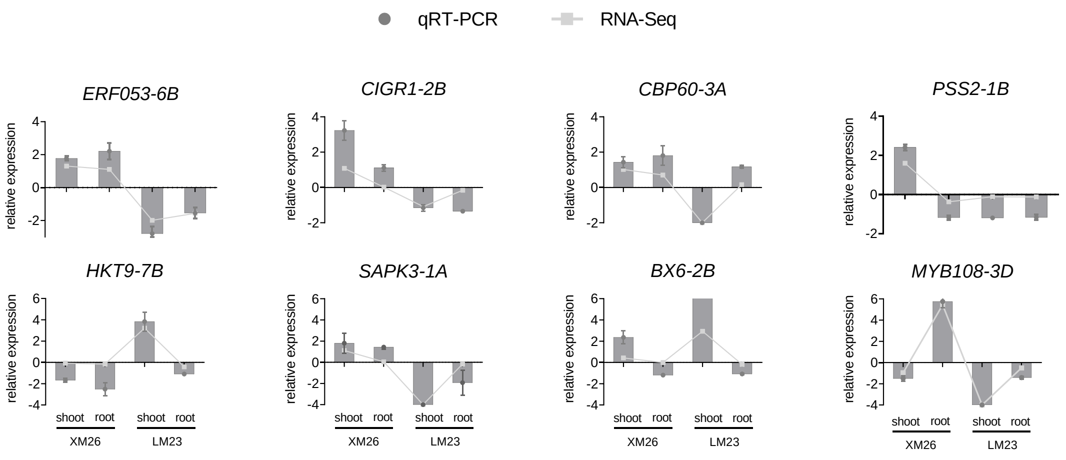

Figure S1. RNA-Seq data accuracy verification. The column represents the relative expression level of qRT-PCR, and the broken line represents the log2(fold change) of RNA-Seq. Positive values on the y axis represent up-regulation of genes, and negative values represent down-regulation of genes.

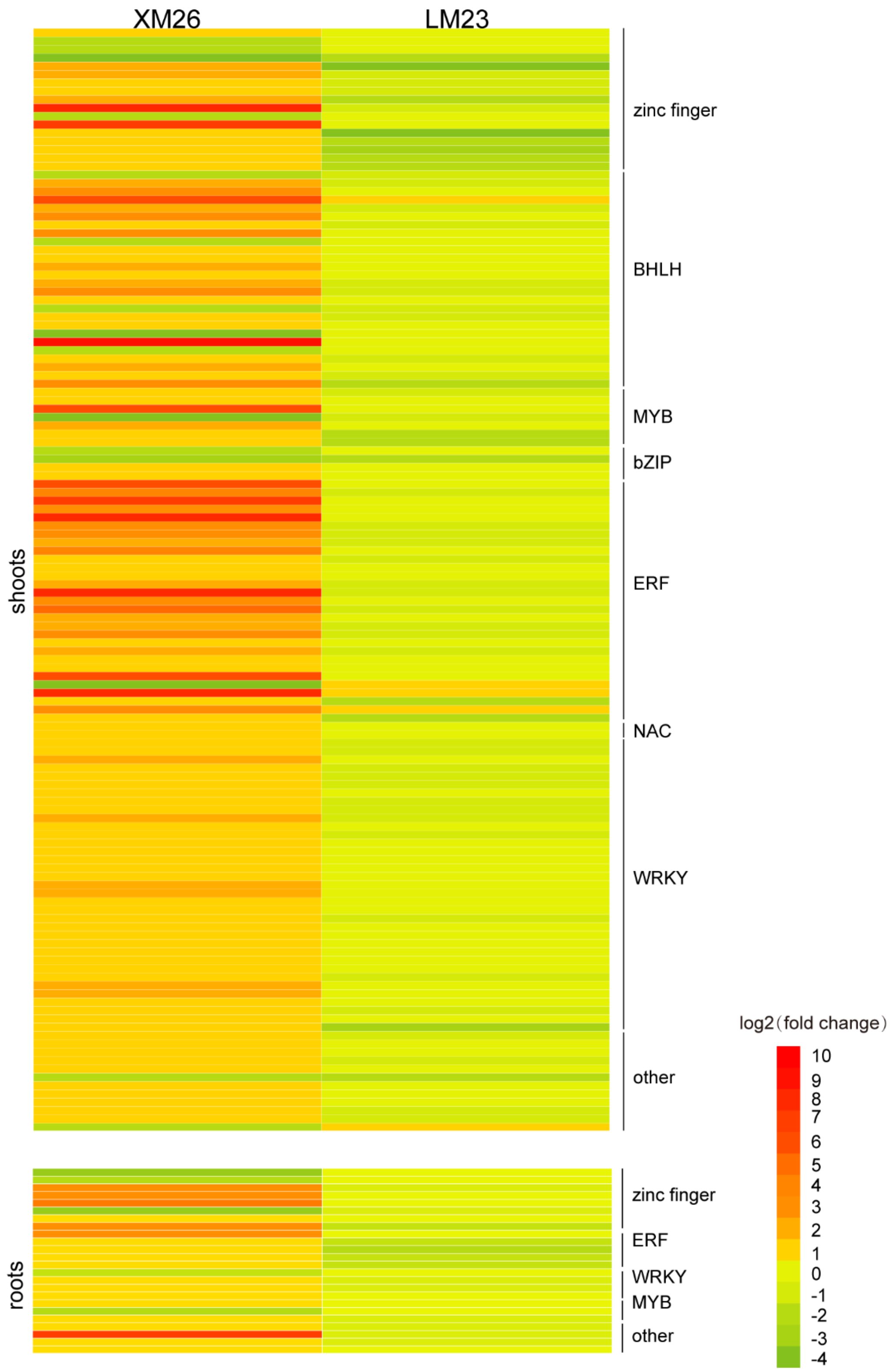

Figure S2. Heatmap expression profile of DEGs involved in transcription factors. The colour scale indicates log2(fold change) gene expression levels. Red indicates high transcript abundance, green indicates low transcript abundance. Right y-axis is different TF families.
